# A rapid phage assay for detection of viable *Mycobacterium avium* subsp. *paratuberculosis* in milk

**DOI:** 10.1038/s41598-021-04451-w

**Published:** 2022-01-10

**Authors:** Sepideh Hosseiniporgham, Lucio Rebechesu, Pierangela Pintore, Stefano Lollai, Maria Dattena, Simone Russo, Angelo Ruiu, Leonardo A. Sechi

**Affiliations:** 1 Dipartimento di Scienze Biomediche, Università di Sassari, Sassari, Italy; 2 Istituto Zooprofilattico della Sardegna, Sassari, Italy; 3 Agenzia Regionale Ricerca in Agricoltura (AGRIS), Bonassai, Italy; 4 National Reference Centre for Paratuberculosis, Sede Territoriale di Piacenza, Istituto Zooprofilattico Sperimentale della Lombardia e dell’Emilia Romagna (IZSLER), Strada Faggiola 1, 29027 Gariga di Podenzano, PC Italy; 5 Istituto Zooprofilattico della Sardegna, Oristano, Italy; 6 Azienda Ospedaliera Universitaria, Sassari, Italy; 7 Mediterranean Center for Disease Control, Sassari, Italy

**Keywords:** Bacteriophages, Infectious-disease diagnostics, Immunology, Microbiology

## Abstract

Paratuberculosis is an incurable gastroenteritis among ruminants that is promoted by *Mycobacterium avium* subsp*. paratuberculosis* (MAP), an acid-fast mycobacterium. To accelerate the detection of viable pathogen, a conventional (peptide mediated magnetic separation: PMS) and novel (phage-bead qPCR: PBQ) phage based assay was optimized. A superior limit of detection (LOD) of 10 MAP per 10 mL milk was suggested for PBQ compared to 100 cells/10 mL for PMS-phage assay. Via PBQ, viable MAP was found in 48.78% out 41 unpasteurized sheep and goat milk samples. Sheep milk samples (n = 29) that were tested by PMS-phage assay contained no viable MAP. The absence of viable MAP in milk collected from 21 of the recent sheep animals was also confirmed by PBQ after a 2-week gap. Although, the two phage assays comparably detected no viable MAP in the milk samples, MAP DNA and antibodies against MAP were recognized in milk and sera of some of these animals within two instances of sampling representing that some sheep animals were MAP shedders. In conclusion, PBQ and PMS-phage could be promising methods for the assessment of MAP viability in milk samples. However, PBQ was privileged over the PMS-phage assay due to the lower LOD, rapidity, higher sensitivity, lack of need to *M. smegmatis* and consequent virucidal treatment that are essential in PMS-phage assay for making lawn and inactivation of exogenous mycobacteriophages respectively.

## Introduction

Paratuberculosis or Johne’s disease (JD) is a chronic gastrointestinal condition among ruminants^1^ that is triggered by *Mycobacterium avium* subsp*. paratuberculosis* (MAP)^2^, an acid fast bacillus with complicated growth requirements^3^. MAP-infected animals frequently excrete the pathogen into milk or the environment via feces and put the susceptible animals at risk of the disease^4,5^. Detection of viable MAP could impede the circulation of the pathogen to new recipients remarkably. Many studies have consensus on the strength of culture as a confirmatory assay for the diagnosis of MAP viability in clinical specimens such as milk, blood, and feces. However, MAP has a lengthy generation time (more than 24 h) that elongates the isolation of bacterium to 7–16 weeks^6–8^. Recently, the selective capture of viable bacteria in milk and other matrixes has been facilitated via biotechnological approaches such as magnetic separation (MS) through purifying the target bacteria and reducing the background signals^9^. Up to now, MS underwent modifications and the capture efficiency of magnetic beads was improved through the application of MAP-specific ligands such as antibodies (monoclonal/polyclonal) and peptides^10–13^. MAP-complementary peptides of aMp3 and aMptD are specific ligands that routinely used in MS for assessment of MAP viability in various samples. aMp3- and aMptD-mediated magnetic beads could retrieve MAP in specimens containing 10^4^ to 10^3^ cfu/mL of the bacterium by 85–100% reducing the rate of cross-reactivity with close mycobacterial species to less than 1%^13^. The functionality of peptide mediated magnetic bead separation (PMS), in isolation and integrated with other confirmatory diagnostic methods such as culture, qPCR IS*900* (Insertion sequence IS*900* is a conserved region that has 16–22 copies in whole MAP genome and is usually targeted in molecular diagnosis of MAP^14–18^), immunoassays (e.g. antigen detection immunoassay), and phage assay^13^ have been evaluated in few studies^19^. Currently, PMS-phage assay introduced a remarkable velocity to the MAP-viability assessments reducing the diagnostic time^8,20–22^ to only 48 h^23^. D29 is a tailed-lytic mycobacteriophage that is frequently applied in PMS-phage assay^24–27^ and it has various mycobacterial hosts such as *M. smegmatis*^26^ *M. chelonae, M. fortuitum*, MAP^28^, *M. tuberculosis*^26^, *M. ulcerans*, *M. scrofulaceum*^28^, and *M. leprae*^29^. Recently, in a modern one-day phage assay (PhMS-qPCR), no more peptides were used as ligands in the capturing structure and mycobacteriophage D29 was directly and covalently (via amine groups) conjugated to the surface of paramagnetic beads. This modification not only improved the limit of detection (LOD_50%_) of MAP via phage assay to only 10 viable cells in 50 mL milk sample, but also reduced the length of procedure to almost 7 h^30^.

The following study aimed to optimize a conventional and novel magnetic separation phage qPCR assay that respectively works with (PMS-phage assay) and without (phage-bead qPCR) intervention of MAP specific complementary peptides of aMp3 and aMptD evaluating the functionality of each assay in detection of viable MAP in sheep and goat milk samples. Moreover, in order to compare the status of viable MAP (absence/presence) in milk samples taken from 21 MAP shedder sheep animals in two instances of sampling, a parallel study via the two phage assays was carried out on sheep milk samples collected within a 2-week gap and the results were compared with other MAP-diagnostic approaches. Later, the specificity and sensitivity of both phage assays were computed through ROC curve analysis.

## Results

### Optimization of phage-bead qPCR (PBQ) in MAP-spiked PBS and milk samples

Phage-bead qPCR (PBQ) could comparably detect viable MAP in both MAP-spiked PBS and milk samples at concentrations between 10^4^ to 10^1^ cfu/mL. As qPCR IS*900* on DNAs extracted from retrieved phage beads depicted that the threshold cycle (TC) corresponding each concentration of MAP in PBS was at the same range as its milk counterpart (Table 1). Since, the lowest detectable concentration of viable MAP DNA in both PBS and milk samples was 10^1^ cfu/mL, this endpoint was adjusted as the limit of detection (LOD) of PBQ in both PBS and milk.

**Table 1:** Optimization of phage-bead qPCR (PBQ) and threshold cycles (TC) corresponding different concentrations of viable MAP DNA in MAP-spiked PBS and a known negative milk sample.

Concentrations (cfu/mL)	PBS (TC)	Milk (TC)
10^4^	32.21	32.56
10^3^	34.21	35.85
10^2^	36.52	39.43
10^1^	40.17	38.24

### PBQ on unpasteurized goat and sheep milk samples in comparison with milk qPCR, milk ELISA, and serum ELISA

**Figure 1:**
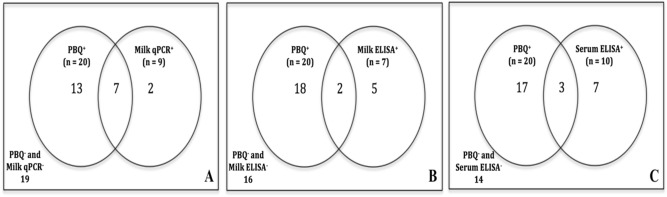
Venn comparison analysis between phage bead qPCR (PBQ) dataset and each MAP diagnostic assay of milk qPCR (**A**), milk ELISA (**B**), and serum ELISA (**C**).

PBQ was assayed on 41 goat and sheep milk samples and MAP DNA belonging to viable cells was detected in 48.78% of samples by qPCR IS*900* at TC between 35.29 to 40.15 cycles corresponding to the concentration of 5.25 × 10^–3^ ng/μL < C > 5.25 × 10^–6^ ng/μL. The result of qPCR IS*900* on DNAs extracted from whole milk samples revealed that only 35% of PBQ-positive cases had detectable concentrations of MAP DNA (C > 5.25 × 10^–6^ ng/μL, TC between 38.82 and 39.9 cycles) corresponding to both dead and viable cells. The level of agreement between PBQ and milk qPCR was estimated fairly significant by 63.41% (kappa = 0.2581, 95% CI = 0.0083 to 0.508) compared to 31.7% and 4.8% that were just PBQ-positive and milk qPCR-positive respectively. In addition, McNemar’s test determined a statistically significant difference in the proportion of PBQ-positive cases rather than milk qPCR-positive subjects [two-tailed *p*-value = 0.0098; Venn analysis (Fig. 1A)].

Further ELISA assessments reveled that among 41 sheep and goat animals 14.63% were both milk and serum ELISA positive, whereas 2.4% and 9.8% of cases were only milk ELISA-positive and serum ELISA-positive respectively. In fact, PBQ assay had almost similar but insignificant level of concordance with both milk and serum ELISA by 43.9% (kappa = − 0.1403, 95% confidence interval (CI) = − 0.3697 to 0.0892) and 41.46% (kappa = − 0.1855, 95% CI = − 0.1855, − 0.4449 to 0.0738) respectively. McNemar between PBQ and milk/serum ELISA confirmed that the proportion of PBQ-positive animals was statistically higher than milk or serum ELISA-positive cases with two-tailed *p*-values of 0.0123 and 0.066 represented by Venn analysis Fig. 1B,C respectively.

**Figure 2:**
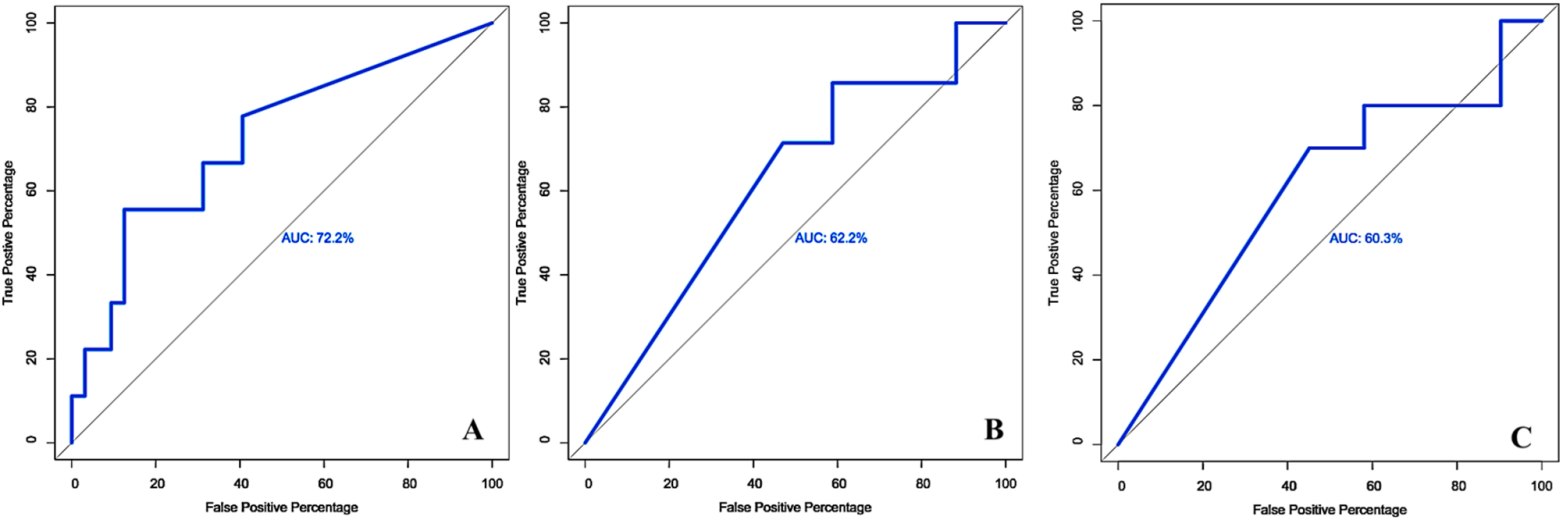
Receiver operating characteristic (ROC) curve analysis and corresponding area under the curve (AUC) analysis on PBQ dataset when milk qPCR (**A**), milk ELISA (**B**), serum ELISA (**C**) were binary reference models.

On the other hand, ROC curve analysis depicted that milk qPCR, as a binary reference model, induced higher specificity to PBQ analysis (AUC = 72.2%, cutoff = 37.935, SP: 87.5%, SN: 55.56%; Fig. 2A) compared to milk and serum ELISA that elevated the sensitivity of PBQ rather than its specificity [milk ELISA (AUC: 62.2%, cutoff: 35.37, SP: 50%, SN: 71.43%; Fig. 2B); serum ELISA (AUC: 60.3%, cutoff: 35.37, SP: 54.84, SN: 70%; Fig. 2C)].

### Optimization of peptide-mediated magnetic separation (PMS) phage assay

**Figure 3:**
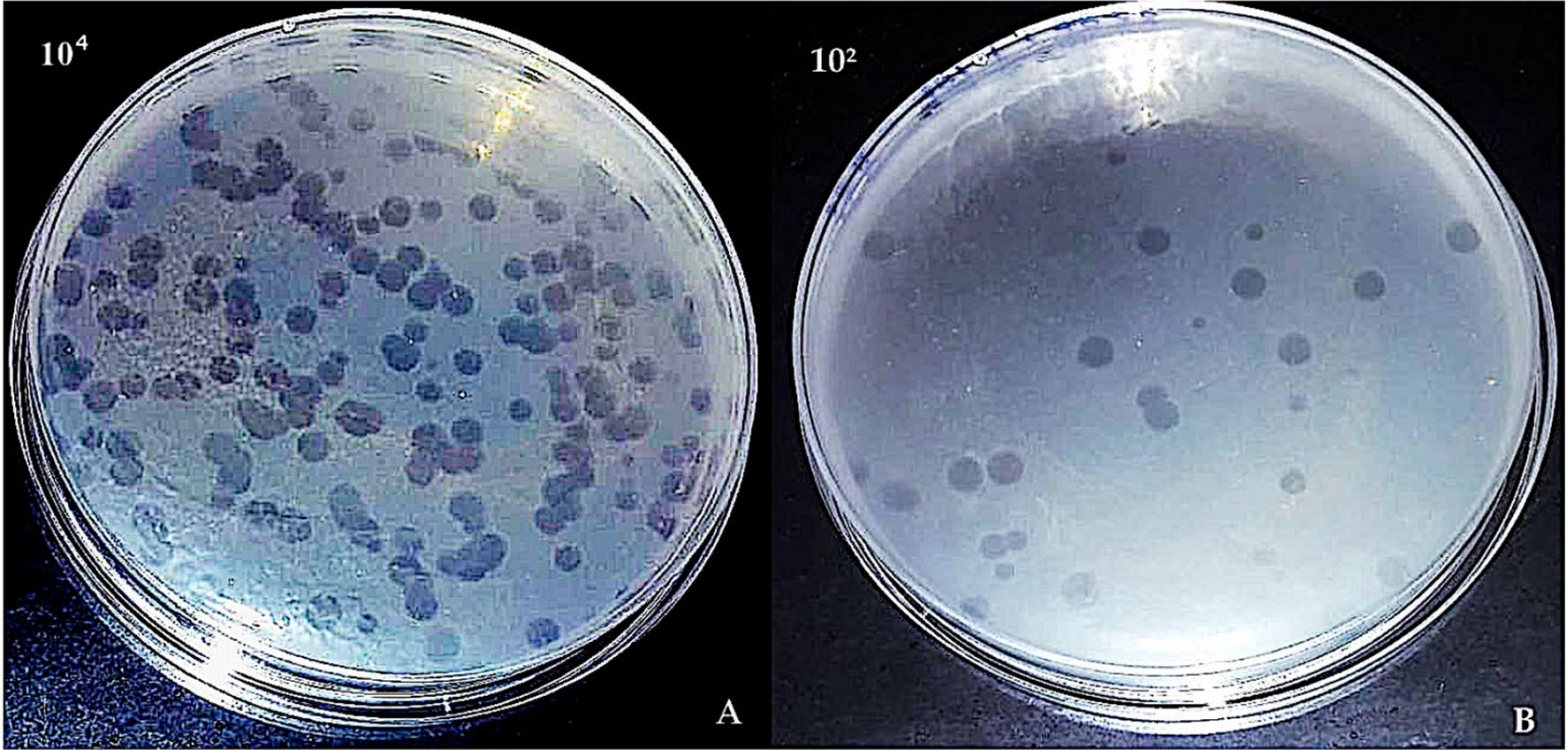
A schematic view of lysed plaques (3–4 nm) on Middlebrook (MB) 7H10 agar corresponding to MAP concentrations of 10^4^ (**A**) and 10^2^ (**B**) cfu/mL in an artificially contaminated milk sample (this photo was taken before the end of the overnight incubation (18-h) at 37 °C).

The result of optimization of PMS-phage assay in artificially MAP contaminated milk samples demonstrated that the numbers of plaques gradually decreased at concentrations between 10^4^ cfu/mL (Fig. 3A) to 10^2^ cfu/mL from many to 30 plaques. Later, DNA was extracted from each 10 plaques located at different zones of the plates and the positivity of DNA was tested by qPCR IS*900* analysis. Since, MAP DNA was discovered in plaques corresponding to the concentration of 10^2^ cfu/mL, the LOD of the assay was adjusted at 10^2^ cfu/mL (Fig. 3B, Table 2).

**Table 2:** Optimization of PMS-phage assay on MAP-spiked milk samples represented by the number of lysed plaques developed on MB 7H10 agar and the positivity status of DNA extracted from lysed plaques via qPCR IS*900* analysis.

Dilutions/concentrations	Number of plaques in milk	qPCR IS*900*
10^−1^/10^6^	Totally lysed	–
10^−2^/10^5^	Totally lysed	–
10^−3^/10^4^	Many	Positive
10^−4^/10^3^	31	Positive
10^−5^/10^2^	30	Positive
10^−6^/10^1^	0	–

### PMS-phage assay on unpasteurized sheep milk samples in comparison with milk qPCR IS*900*, milk ELISA, and serum ELISA

**Figure 4:**
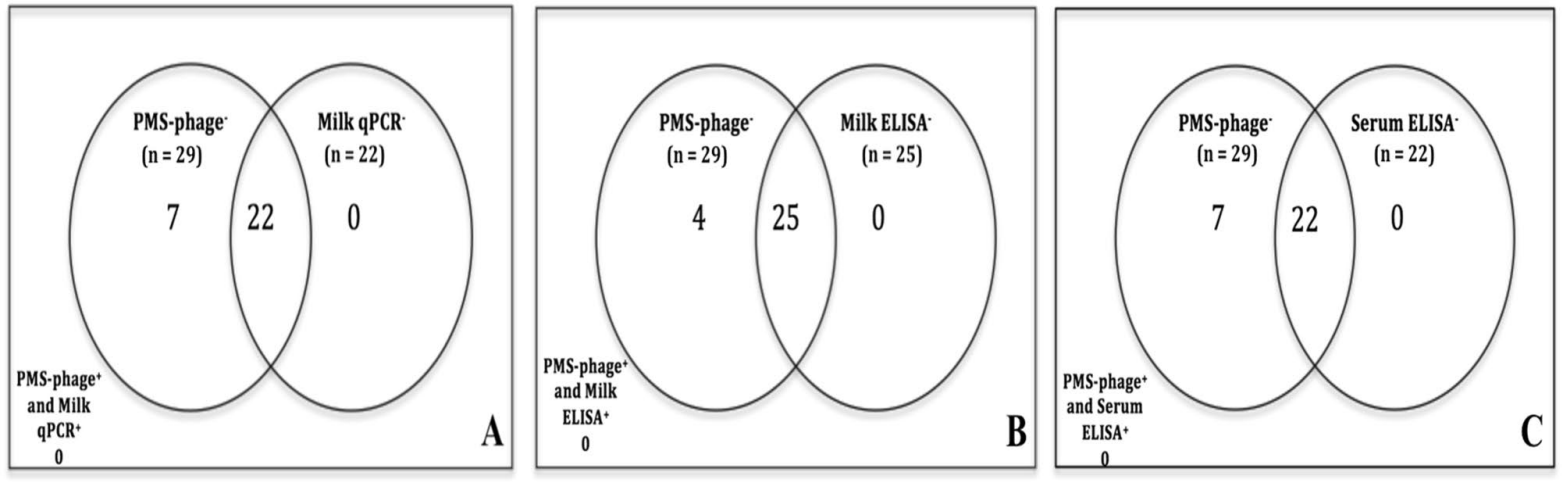
Venn comparison analysis between peptide mediated magnetic separation phage assay (PMS-phage) dataset and each MAP diagnostic assay of milk qPCR (**A**), milk ELISA (**B**), and serum ELISA (**C**).

Despite the fact that some samples produced IS*900*-negative lysed plaques, no viable MAP was detected in 29 unpasteurized milk samples collected from asymptomatic sheep animals via PMS-phage assay (plaque numbers ranged from 1 to above 400 pfu/10 mL). However, milk qPCR IS*900* on DNAs extracted from whole milk samples revealed that 24.14% (7 out of 29) of the samples contained traces of MAP DNA at threshold cycles (TC) between 37.16 to 41.07 cycles (C > 5.25 × 10^–6^ ng/μL). Surprisingly, PMS-phage assay and milk qPCR were concordant by 75.86% (kappa = 0) due to the reason that many cases were detected negative by both tests. On the other hand, 13.79% and 24.14% out of 29 animals were just milk and serum ELISA-positive respectively. Moreover, PMS-phage assay was strongly concordant with milk and serum ELISA (both with kappa value of zero) by 86.21% and 75.86%. McNemar’s test was significant between PMS-phage and milk qPCR/serum ELISA representing that the proportion of MAP negative cases detected by each bi-test of PMS-phage and milk qPCR, PMS-phage and serum ELISA was statistically significant (both with two-tailed *p*-value of 0.0233; Venn analysis Fig. 4A,C) rather than milk qPCR-positive and serum ELISA-positive cases. In contrast, McNemar was insignificant between PMS-phage and milk ELISA (two-tailed *p*-value = 0.1336; Fig. 4B). This is under the condition that the number of cases that was negative by both PMS-phage and milk ELISA was predominant (86.21%) among other groups. Further ROC curve analysis represented that PMS-phage assay was extremely specific and insensitive when milk qPCR, milk ELISA, and serum ELISA were reference models (SP: 1, SN: 0). This is due to the fact that none of samples contained viable MAP that could induce remarkable differences to the ROC curve analysis.

### PBQ and PMS-phage assay on milk samples taken from MAP shedder sheep animals in two instances of sampling in comparison with milk qPCR, milk ELISA, and serum ELISA

**Figure 5:**
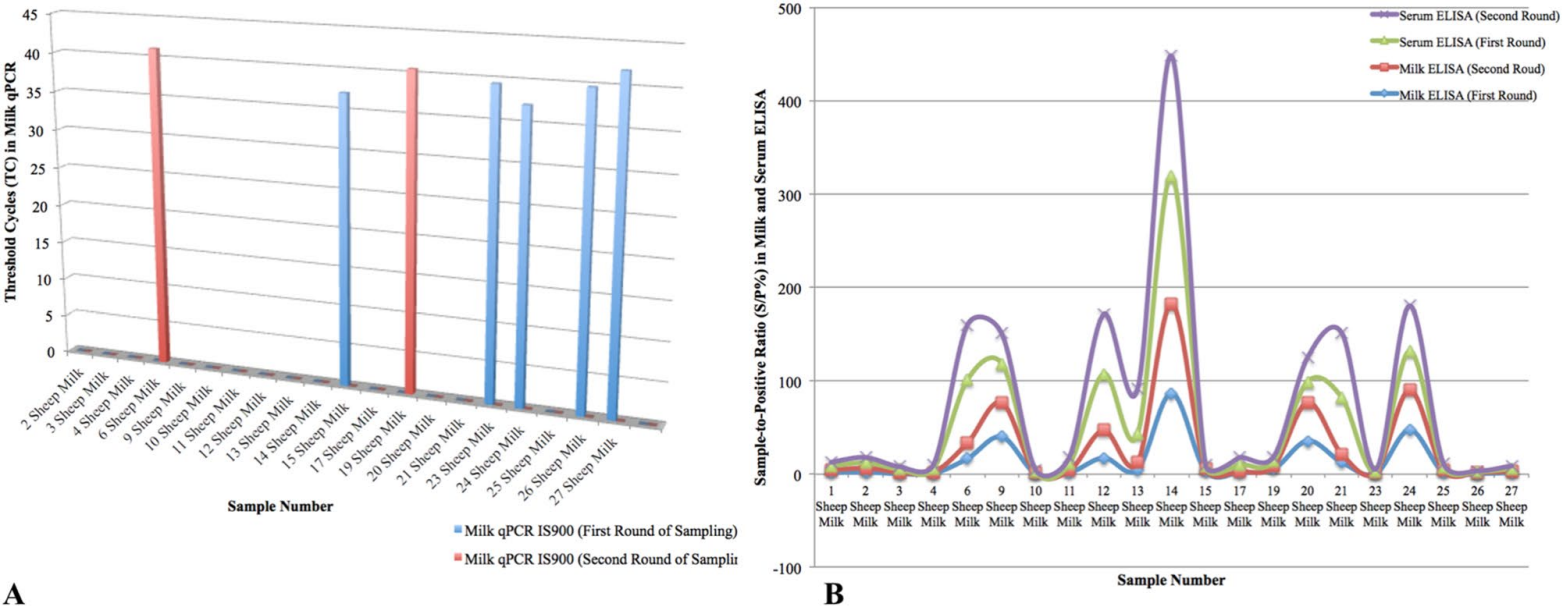
Comparison among the results of three diagnostic assays of milk qPCR, milk ELISA, serum ELISA at the first and second rounds of sampling. This comparison is based on threshold cycles (TC) and sample-to-positive ratio (S/P%) generated in qPCR IS*900* (**A**) and milk/serum ELISA (**B**) respectively.

The viability of MAP was confirmed in none of 42 sheep milk samples that were tested by both PBQ and PMS-phage assays. These samples were collected from 21 sheep animals within a 2-week gap. qPCR IS*900* on DNAs extracted from the same milk samples depicted that some animals were intermittently shedding MAP into milk, in which 23.8% of samples that were firstly milk qPCR-positive became negative at the second time, instead 9.5% of samples (n = 2) that were negative at first, became positive at the second time (Fig. 5A). Furthermore, ELISA on milk and serum samples (each n = 42) taken from the same animals in two rounds of sampling revealed that 19.04% and 33.33% of animals were respectively milk- and serum ELISA-positive at both rounds, and only one sheep that was firstly milk ELISA-negative became positive at the second round (Fig. 5B). Although the titers of antibody directed against MAP in milk and serum samples saw a gradual rise or fall by 0–18.04 degrees in sample-to-positive ratio (S/P%) in 2-week gap, milk and serum ELISA were highly correlated and both of them had similar progress of changes during the course of study (Fig. 5B).

## Discussion

The slow-growth characteristic of MAP imposed huge struggles on the detection of viable bacterium through culture-based analysis. Up to now, many studies have been conducted representing that selective capture of target bacteria via magnetic beads coated with MAP-specific ligands along with a phage amplification step could speed up the discovery of viable MAP in various samples significantly. However, there is still room for improvement of the technique not only by reducing the length of the procedure but also by enhancing the specificity and sensitivity of the assay coincidently. Recently, in a novel phage-based assay the procedures of capture and subsequent infection of viable MAP (existed in milk samples) were integrated into one step using magnetic beads that directly coated with mycobacteriophage D29^30^. Accordingly, we were inspired to optimize this novel technique along with a conventional phage assay that worked with intervention of peptides of aMp3 and aMptD and evaluate the functionality of the two methods on sheep and goat milk samples. aMp3 and aMptD are biotinylated MAP specific complementary peptides that were recognized through phage display biopanning against whole MAP cell and the MAP-surface exposed protein of aMptD (that is regulated by MAP–specific ABC Transporter operon (mpt)) respectively^5,11^. Furthermore, mycobacteriophage D29, a lytic phage, is commonly used in phage amplification studies in order to infect the viable MAP cells and liberate DNAs from the recovered cells. Although, D29 is not a specific phage for only MAP^28,31,32^, it specifically expresses its DNA in its viable mycobacterial hosts^33^.

In optimization of the two phage-based assays in this study, PBQ excelled PMS-phage assay by tenfold in detection of the lowest quantity of MAP (10^1^ cfu/10 mL) existed in an artificially MAP spiked milk sample. This functionality was previously reported by the original phage-bead qPCR study on assessment of MAP viability in bovine milk samples^30^, although the tenfold reduction in the LOD of PMS-phage assay was possibly due to the sample’s final volume to which the assay was optimized for (10 mL compared to 50 mL in other studies^34^). Up to now, PMS-phage assay was tested on various specimens such as raw milk (LOD_50%_ = 0.90–0.95), blood, and peripheral blood mononuclear cells (PBMC) diagnosing MAP at concentrations between 7.3 × 10^2^ pfu/mL to 10 MAP cells/50 mL milk^34–36^. Interestingly, a survey on MAP-spiked milk samples depicted that heat treatment before PMS-phage assay could not affect the MAP viability as well as phage functionality, in which a significant correlation was found between cfu/mL and pfu/mL in unheated (r^2^ = 0.943) and heated (r^2^ = 0.971) milk samples^19^. Previously, PMS-phage assay was privileged compared to PMS-PCR and PMS-MGIT in detection of viable MAP in individual and bulk tank milk (BTM) samples^34^.

To the best of our knowledge, LOD in PMS-phage assay might be affected by several factors such as the final volume of sample and the lipid content of milk samples.

Our PBQ assay was examined on 41 unpasteurized goat and sheep milk samples indicating that various concentrations of viable MAP existed in 48.78% of the specimens. Recently, a study on the efficiency of phage-bead qPCR on BTMs collected from 100 Northern Ireland dairy farms depicted that 49% of milk samples contained various concentrations of viable MAP between 3 to 126 cells in a final volume of 50 mL^30^. We found that PBQ had the highest level of agreement with milk qPCR by 63.41% compared to 43.9% concordance between PBQ and milk/serum ELISA. This consensus could even be noticed in ROC curve analysis, in which a significant specificity (SP: 87.5%) and moderate sensitivity (SN: 55.56%) were induced to PBQ analysis when the binary reference model was adjusted to milk qPCR compared to the condition that milk ELISA (SP: 50%, SN: 71.43%) and serum ELISA (SP: 54.84, SN: 70%) were independently a gold standard and the sensitivity of PBQ enhanced by 70%, whereas its specificity dropped by 50%. Furthermore, no viable MAP was detected in 29 sheep milk samples that were tested by PMS-phage assay. However, some samples developed lysed plaques that were qPCR IS*900*-negative. This might be the consequence of either disability of the virucide (ferrous ammonium sulfate: FAS) in inactivation of all exogenous mycobacteriophages or attachment of mycobacterial species other than MAP to the surface of peptide mediated magnetic beads. According to our hypothesis, mycobacterial species other than MAP might be trapped in the lipid-enriched structure of sheep milk, infected with mycobacteriophage, and carried over to the culture step.

However, PMS-phage assay was remarkably in agreement with other MAP diagnostic assays including milk qPCR (75.86%), milk ELISA (86.21%), and serum ELISA (75.86%). This is due to the fact that the majority of cases were detected negative by each bi-test of “PMS phage and milk qPCR”, “PMS phage and milk ELISA”, and “PMS phage and serum ELISA”. Further ROC curve analysis on PMS-phage assay data depicted that none of the selected reference models could influence sensitivity of PMS-phage assay significantly. In which PMS-phage assay had the utmost specificity (100%) but least sensitivity (0%) with the all gold standard models. This is definitely due to the small sample size and the absence of viable MAP in all samples tested by PMS-phage assay that could impose measurable differences to the statistical analysis. Similarly, a comparative study on evaluation of PMS-phage assay and PMS-culture for detection of viable MAP in bovine milk samples demonstrated that both PMS-based methods had a noticeable specificity (100% and 96.2% resp.) rather than sensitivity (32.5% and 25% resp.), since the level of concordance between the two assays was only 8%^37^.

Our parallel study via the two phage assays on sheep milk sample collected from intermittent MAP-shedder animals in two instances of sampling depicted that PBQ and PMS-phage assays comparably detected no viable MAP in these milk samples. In fact, the first and second rounds of samples were tested by PMS-phage assay and PBQ respectively, since we did not have access to sufficient amount of milk to test all of them by the two phage assays at the same time. However, the two times sampling provided us with a double opportunity to compare the positivity status (via DNA/antibody) of the participated animals by other MAP diagnostic assays. Surprisingly, we noticed that some of the tested animals were asymptomatic cases that suffered from a progressive Johne’s disease, since the positivity status of them changed either by milk qPCR analysis or milk/serum ELISA within the course of study. In which, the antibody titers against MAP in milk and serum samples collected from these animals fluctuated between 0–18.04 degrees in S/P% in a 2-week gap. This is under the condition that milk and serum ELISA were favorably in agreement, in which both assays had similar patterns of changes in sample-to-positive ratio during the course of study.

Our findings suggest that PBQ and PMS-phage could be promising methods for assessment of MAP viability in milk and even other clinical samples. PBQ detected viable MAP among numbers of unpasteurized goat and sheep milk samples that collected from apparently healthy animals. PBQ had a considerable level of concordance with milk qPCR. The conformity between PBQ and milk qPCR could even be seen in ROC curve analysis, in which milk qPCR as a reference model induced a remarkable specificity and sensitivity to PBQ. Our comparative study via the two phage assays on sheep milk samples taken from the same animals in two instances of sampling disclosed that both phage assays functioned comparably, however we noticed that some PBQ/PMS negative animals were intermit MAP shedders, in which animals that were firstly milk qPCR-positive, turned negative at the second time or vice versa. This heterogeneity in the result of milk qPCR analysis might be the consequence of diversity of animals in the stages of Johne’s disease. This claim has been proved through a follow-up ELISA test on milk and serum samples taken from the same MAP shedder animals revealing that the antibody titers against MAP in milk and serum modified within the two times sampling. Eventually, in this study, PBQ overprivileged PMS-phage assay due to three factors including: (1) lower LOD: PBQ with LOD of 10 viable MAP cells in 10 mL milk excelled PMS-phage assay by 10 folds; (2) rapidity and cost effectiveness: PBQ could be carried out with minimum requirements between 7–9 h, whereas PMS-phage assay has a longer procedure of at least 48 h due to the need to an additional culture step with *M. smegmatis* for visualization of lysed plaques; (3) lack of need for virocidal treatment: based on our experience the quality of virocide (FAS) could influence the number of lysed plaques in PMS-phage assay and this could undermine the specificity of the assay as well. This is under the condition that no virocidal treatment is needed in PBQ.

## Materials and methods

### Bacterial strains

*Mycobacterium avium* subsp. *paratuberculosis* (MAP) strain CR131 (ATCC 19698) and *Mycobacterium avium* subsp. *paratuberculosis* strain 1515 (ATCC 43015) were used as positive controls throughout the study. *Mycobacterium smegmatis* strain MC^2^155 (ATCC 700084) was also used as a negative mycobacterial species to make a lawn for visualization of lysed plaques in peptide mediated magnetic separation phage assay (PMS-phage). The mycobacterial strains were grown in Middlebrook 7H9 broth (MB; Sigma-Aldrich, Milan, Italy) supplemented with 10% Oleic Albumin Dextrose Catalase (OADC; Sigma-Aldrich, Milan, Italy) and incubated at 37 °C for 3 days to 4 weeks (depending on the mycobacterial species). To accelerate the growth rate of MAP, Mycobactin J (2 mg; Allied Monitor, Fayette, MO, USA) as an iron-chelated growth factor was just added into MAP culture^14,38^.

### Phage strain

The mycobacteriophage D29 strain that was used throughout this study came with a commercial phage assay kit (Actiphage, UK, England) and was characterized with the plaque size of 3–4 mm. To reach the working concentration of 10^9^ pfu/mL, the phage was propagated as described before^30^ and stored at 4 °C until use.

### Sample collection

The studied sheep and goat animals were selected from research herds belonging to Agenzia Regionale Ricerca in Agricoltura (AGRIS; Bonassai, Sardinia, Italy) and all methods were performed in accordance with relevant guidelines and regulations legislated by the Institute of Zooprofilattico in Sardinia, Italy (Protocol Number: 0005147/17). Accordingly, a total of 70 unpasteurized sheep and goat milk samples were collected from asymptomatic animals (Table 3). The samples were taken in a sterile condition, kept at 4 °C during transportation. The aliquots that were subjected to phage assay stored at 4 °C overnight to be tested a day after. Blood samples were also drained from jugular veins of the same animals, dispensed into sodium heparin Vacutainer tubes (Becton Dickinson (BD), Milan, Italy), transferred to the diagnostic laboratory at RT, and underwent the process of serum separation.

**Table 3:** Number of unpasteurized milk and blood samples and their animal sources.

Number of samples/times of sampling	Type of sample
20/once	Milk and blood samples from asymptomatic goats
8/once	Milk and blood samples from asymptomatic sheep
21/twice	Milk and blood samples from asymptomatic sheep

### Preparation of phage D29-beads

Mycobacteriophage D29 was used as ligand for coating magnetic beads as previously descried by Foddai and Grant^30^ in their recent publication. This attachment was fortified by covalent bonds between free amines on the surface of bacteriophage and tosyl groups on Tosylactivated magnetic beads. Briefly, 10 mg Dynabeads MyOne Tosylactivated (Thermo Fisher, code 65501, Milan, Italy) were transferred into Eppendorf (2 mL), washed two times with 1 mL sodium carbonate decahydrate buffer (VWR, Milan, Italy) at concentration of 0.1 M and pH = 9.5 as binding buffer, and magnetically recovered. Then, beads were resuspended at the same binding buffer, exposed to mycobacteriophage D29 (Actiphage, UK, England) at concentration of 10^8^ pfu/mL, and incubated at 37 °C overnight rotating (10–20 rpm) continually. A day after, beads were recovered magnetically, washed two times with the binding buffer, and resuspended in Middlebrook 7H9 medium supplemented with 10% OADC and 2 mM CaCl_2_. At the end, the phage-bead suspension was stored at 4 °C and only fifteen minutes prior inoculation transferred into the room temperature in order to be equilibrated with this temperature.

### Optimization and evaluation of phage beads qPCR for detection of viable MAP in artificially MAP-contaminated and unpasteurized milk samples

MAP suspension (CR131 strain) at stationary phase was de-clumped using sonicator (Elmasonic S 30 (H), Singen, Germany) at 37 kHz and 10 °C for 4 min and its concentration was adjusted at optical density of 0.8 (10^8^ cfu/mL) at 600 nm. Then, this stuck was serially diluted in 8 folds and 1 mL of dilutions containing 10^4^ to 10^1^ cfu/mL were used for spiking a known negative commercial bovine milk sample (9 mL). Artificially contaminated milk samples were mixed thoroughly, incubated at 37 °C for 30 min, and centrifuged at 2500×*g* and 4 °C for 15 min consequently. To monitor the functionality of the assay and prevent any carry-over, MAP standard strains of CR131/1515 and phosphate-buffered saline (PBS) were respectively applied as positive and negative controls from the beginning of each assessment. Later, supernatant containing cream and whey were decanted, and pellet was resuspended in 1 mL MB 7H9 (supplemented with 10% OADC and 2 mM CaCl_2_). At the next step, phage beads (15 μL) were added to each sample and incubated at 37 °C while rotating (10–20 rpm) for 30 min. After two washing steps with 1 mL PBS-Tween 20 (PBST; 0.05%), beads were magnetically recovered, resuspended in 50 μL MB 7H9 (supplemented with 10% OADC and 2 mM CaCl_2_), and incubated at 37 °C for 2 h without agitation. Then, samples were heat-shocked at 55 °C for 2 min and centrifuged at 10,000×*g* and RT for 1 min. At the end, supernatant containing DNA was aspirated and transferred into new PCR micro tubes and stored at 4 °C for short time or − 20 °C for longer period. Regarding the unpasteurized milk samples, 10 mL of each sample were incubated at RT for 1 h and 37 °C for 30 min respectively. Then, samples underwent the same steps as optimization procedure.

### Preparation of peptides-mediated magnetic beads using two MAP-complementary peptides of aMp3 and aMptD

Dynabeads MyOne Tosylactivated (Thermo Fisher, Life Technologies, Milan, Italy, Code 65501) were separately coated with MAP-complementary peptides of aMp3 and aMptD according to the manufacture’s instruction as described before^34^. Briefly, 250 μL of the beads were washed twice with Sodium Borate 0.1 M (as coating buffer; pH = 9.5), and resuspended in 100 μL of the same buffer. Then, aMp3 or aMptD peptides (Table 4) were immobilized (0.25 μg/mL^39^) on the retrieved beads and coating buffer was added to this suspension up to the final volume of 895 μL. Later, ammonium sulfate 3 M (415 μL) was added to this mix, vortexed thoroughly, and this was followed by the incubation of suspension at 37 °C overnight while rotating (10–20 rpm) continually. A day after, the coated beads were washed twice with 1 mL 1 × PBS and resuspended in 500 μL 1 × PBS (without blocking buffer). Eventually, the capture beads were stored at 4 °C until use.

**Table 4:** Capture peptides used for coating Dynabeads MyOne Tosylactivated and their sequences.

Peptide name	Sequence
aMp3	NYVIHDVPRHPA^40^
aMptD	GHNHHHQHHRPQ^5^

### Optimization and evaluation of peptide-mediated magnetic separation (PMS) phage assay for detection of viable MAP in artificially MAP-contaminated and unpasteurized milk samples

Aliquots of a known-MAP-negative commercial bovine milk sample (9 mL) were spiked with different concentrations of MAP stain CR131 (1 mL; the optical density of stock was adjusted at 0.2 corresponding the concentration of 10^7^ cfu/mL), vortexed, and incubated at 37 °C for 30 min. Then, samples were centrifuged at 2500×*g* and 4 °C for 15 min, cream and whey phases were carefully decanted, pellet was resuspended in 1 mL Middlebrook 7H9 broth and sonicated at 37 kHz and 10 °C for 4 min. Negative and positive controls were included from the initial step as mentioned before. Then, samples were transferred into Eppendorfs (2 mL) and 5 μL of each type of magnetic beads coated with aMptD or aMp3 were added to each sample. Later, samples were agitated for 30 min by rotator (Stuart) at RT and placed on a magnetic rack for 10 min. Subsequently, liquid was aspirated and MAP-captured beads were washed three times with PBS-Tween 20 (1 mL; 0.05%) while each magnetic separation step took 2 min. The retrieved beads were resuspended in 1 mL MB 7H9 (contained 10% OADC and 2 mM CaCl_2_), transferred into flip-top vials, and incubated at 37 °C overnight (18 h). A day after, captured MAP cells were infected by mycobacteriophage D29 at final concentration of 10^8^ cfu/mL (100 μL) and samples were incubated at 37 °C for another 2 h. Later, exogenous phages were inactivated via adding 100 μL ferrous ammonium sulfate (FAS; 100 mM) to each sample following a continues rotation step at RT for 10 min. Consequently, FAS was neutralized adding 5 mL MB 7H9 (contained 10% OADC and 2 mM CaCl_2_) and samples were incubated at 37 °C for 90 min. To form a lawn around the lysed plaques that each corresponding to a phage-infected mycobacterium, 1 mL *M. smegmatis* at concentration of 10^8^ cfu/mL (OD = 0.7–1) was added to each sample. Eventually, the suspensions were cultured in petri dishes with 5 mL MB 7H10 agar (55 °C), allowed to solidify at RT for 30 min, and incubated at 37 °C overnight (18 h).

In regard to unpasteurized sheep milk samples, milk aliquots (10 mL) incubated at RT/1 h and 37 °C/30 min respectively, and assayed as described in optimization step.

### DNA extraction from lysed plaques and milk samples

Lysed plaques appeared on plates after 18 h incubation at 37 °C and DNA was extracted from them using freeze-squeeze method^41^. In which, plaques developed on MB 7H10 agar were excised (1–10) and were placed on a 200 μL-filter tip fitted in a 1.5 mL microcentrifuge tube. Then, tubes were stored at − 80 °C for 5 min and immediately centrifuged at highest speed (16,000×*g*) for 3 min. The filtrate was transferred into a new microcentrifuge tube and stored at − 28 °C to be analyzed by qPCR.

Moreover, cream and pellet parts of each milk sample were harvested and treated with 0.75% w/v hexadecylpyridinium chloride (HPC; Sigma-Aldrich, Milan, Italy) as described before^14,38^ and DNA was extracted from both fractions using Norgen Kit (Norgen Biotek Corp., Thorold, ON, Canada) according to the instruction described for the extraction of DNA from unknown or gram positive bacteria.

### Real-time quantitative (qPCR) analysis on DNAs extracted from milk, phage-beads, and plaques in phage assays

Prior to any analyses on real samples, qPCR was standardized based on the selected set of primers and prob. For this purpose, MAP genome copy number was computed via following formula^42,43^:

$${\text{MAP}}\;{\text{ genome }}\;{\text{copy }}\;{\text{number}} = \left( {{\text{Amount }}\;{\text{of}}\;{\text{ DNA}}\; \, \left( {{\text{ng}}} \right) \times {\text{Avogadro's }}\;{\text{number}}} \right) \div ({\text{Length }}\;{\text{of }}\;{\text{MAP}}\;{\text{ DNA}} \times {\text{Convention }}\;{\text{factor}} \times {\text{Average }}\;{\text{mass }}\;{\text{of}}\; \, 1{\text{-bp }}\;{\text{of}}\;{\text{ DNA}}).$$

Avogadro’s number, length of MAP DNA, convention factor, and average mass of 1-bp of DNA were considered 6.062 × 10^23^ mol^−1^, 4,829,781-bp, 1 × 10^9^ ng/g, and 660 g/mol respectively^42,43^. The number of MAP genome copies in one microliter of DNA MAP stock (ATCC 43015) was adjusted at 10^7^ copies/μL. Accordingly, the stock DNA was serially diluted in 10 folds and qPCR standard curve analysis was carried out (Correlation coefficient: 1.00, Slop value: − 3.603, PCR efficiency: 89.5%) (Table 5).

**Table 5:** Standardization of qPCR IS*900* analysis and threshold cycles (TC) corresponding to numbers of MAP copies in each reaction.

Copy numbers of dna template (copies/μL)	Threshold cycle (TC) in qPCR IS*900*
10^7^	18.5
10^6^	22.3
10^5^	26
10^4^	29.5
10^3^	33
10^2^	36.3
10^1^	37.4
1	0
0.1	0
0.01	0

In another step, a fragment at length of 67-bp^42^ in insertion sequence 900 (IS*900*) was targeted in DNAs extracted from milk/plaques (resulted by PMS-phage assay)/supernatants (resulted by PBQ) and amplified by qPCR using QuantStudio 7 Flex System (Thermo Fisher Scientific, Applied Biosystems) and the data was analyzed by QuantStudio Real-Time PCR Software v1.3 (Thermo Fisher Scientific, Applied Biosystems). In general, each qPCR reaction contained 10 μL master mix 2 × (QuantiFast Probe PCR Kits, Milan, Italy), 0.3 μM of each primer of IS*900*-F and IS*900*-R (Table 5), 0.15 μM IS*900* TaqMan prob (labeled with FAM; Table 6), 0.2 exogenous DNA as internal amplification control 0.5 × (IAC), 1 μL mixed primer and probe for IAC 0.5 × (labeled with VIC), 20–100 ng (4 μL) of DNA template, and PCR grade water up to the final volume of 20 μL. Positive (*Mycobacterium avium* subsp. *paratuberculosis* strain 1515) and negative controls (water and Master Mix) were assigned in all qPCR analyses. Accordingly, the amplification was performed under the following condition: initial denaturation at 95 °C for 15 min, followed by 50 cycles of denaturation at 95 °C for 15 s and annealing at 60 °C for 1 min.

**Table 6:** Primers, probe, and their sequences used in qPCR analysis.

Primers and probe	Sequences
IS*900*-F	5′-CCGGTAAGGCCGACCATTA-3′
IS*900*-R	5′-ACCCGCTGCGAGAGCA-3′
IS*900* probe	6FAM-CATGGTTATTAACGACGACGCGCAGC-TAMRA

In qPCR analyses, TC values of below and around 40 were considered positive, since in optimization of PBQ assay the TC value corresponding to the lowest detectable concentration of MAP DNA (10^1^) in 10 mL of sample was 40.17 cycles (Table 1).

### Milk and serum ELISA

After receiving blood samples in diagnostic laboratory, samples were incubated at RT for 1 h to be settled down. Then, they were centrifuged at 2500 rpm and 4 °C for 15 min; serum was separated from blood cells and stored at − 28 °C.

In the meantime, aliquots of milk samples (1 mL) that were subjected to ELISA were centrifuged at 10,000×*g* and 4 °C for 2 min^38^, whey phase (the liquid between cream and pellet) was aspirated in new micro tubes, and stored at − 28 °C until use.

Later, the titers of antibodies directed against MAP in milk and serum samples were evaluated by an indirect commercial ELISA kit for diagnosis of Paratuberculosis (IDEXX Laboratories, Westbrook, ME, USA) according to the manufacture instruction as described before^14,38^. Eventually, the optical density (OD) of each sample was converted into sample-to-positive ratio (S/P%) and interpreted as follows: the S/P% below 20, between 20–30, and 30 and above corresponded to negative, suspect, and positive values respectively.

### Statistical analysis

Receiver operating characteristic (ROC) and area under the curve (AUC) were carried out via R software (version 4.0.5) and the sensitivity and specificity of phage-beads qPCR (PBQ) and peptide-mediated-magnetic separation (PMS) phage assays were evaluated at different cutoffs using various binary reference models including milk qPCR, milk ELISA, and serum ELISA. The level of dependency between PBQ/PMS-phage assay and other MAP diagnostic assays was computed using kappa co-efficient on GraphPad Prism while statistical significance was adjusted for a *p*-value of < 0.05. Additionally, McNemar’s test for paired data (Online GraphPad Prism Software) and Venn analysis were performed in order to estimate and represent the distribution of samples based on positivity/negativity status in each binary test of PBQ/PMS-phage with milk qPCR, PBQ/PMS-phage with milk ELISA, and PBQ/PMS-phage with serum ELISA.

### Ethics declarations

All animal procedures in this study were reviewed and approved by the ethics committee of the Institute of Zooprofilattico in Sardinia, Italy (Protocol Number: 0005147/17), and the authors complied with the ARRIVE guidelines.
